# Fate Mapping for Activation-Induced Cytidine Deaminase (AID) Marks Non-Lymphoid Cells During Mouse Development

**DOI:** 10.1371/journal.pone.0069208

**Published:** 2013-07-08

**Authors:** Philipp C. Rommel, David Bosque, Alexander D. Gitlin, Gist F. Croft, Nathaniel Heintz, Rafael Casellas, Michel C. Nussenzweig, Skirmantas Kriaucionis, Davide F. Robbiani

**Affiliations:** 1 Laboratory of Molecular Immunology, the Rockefeller University, New York, New York, United States of America; 2 Laboratory of Molecular Vertebrate Embryology, The Rockefeller University, New York, New York, United States of America; 3 Laboratory of Molecular Biology, The Rockefeller University, New York, New York, United States of America; 4 Howard Hughes Medical Institute, The Rockefeller University, New York, New York, United States of America; 5 Genomics and Immunity, NIAMS, and Center for Cancer Research, NCI, National Institutes of Health, Bethesda, Maryland, United States of America; 6 Ludwig Institute for Cancer Research and University of Oxford, Oxford, United Kingdom; Chang Gung University, Taiwan

## Abstract

The *Aicda* gene encodes Activation-Induced cytidine Deaminase (AID), an enzyme essential for remodeling antibody genes in mature B lymphocytes. AID is also responsible for DNA damage at oncogenes, leading to their mutation and cancer-associated chromosome translocation in lymphoma. We used fate mapping and AID^GFP^ reporter mice to determine if AID expression in the mouse extends beyond lymphocytes. We discovered that AID^cre^ tags a small fraction of non-lymphoid cells starting at 10.5 days post conception (dpc), and that AID^GFP+^ cells are detectable at dpc 11.5 and 12.5. Embryonic cells are tagged by AID^cre^ in the submandibular region, where conditional deletion of the tumor suppressor PTEN causes squamous papillomas. AID^cre^ also tags non-lymphoid cells in the embryonic central nervous system. Finally, in the adult mouse brain, AID^cre^ marks a small fraction of diverse neurons and distinct neuronal populations, including pyramidal cells in cortical layer IV.

## Introduction

Activation-induced cytidine deaminase (AID) is required for somatic hypermutation (SHM) and class switch recombination (CSR), two DNA diversification reactions of mature B lymphocytes. Upon activation in response to antigen, B cells proliferate, form germinal centers (specialized anatomical structures within lymphoid organs), and express high levels of AID [1]. At antibody (immunoglobulin) genes, AID deaminates cytosines into uracils on single-stranded DNA during transcription. The resulting uracil-guanine mismatch can be processed in many ways, leading to DNA mutations (SHM) and DNA double-strand breaks (an obligate intermediate during CSR). As a result, antibodies are generated with higher affinity against the antigen, and with distinct effector functions, such as the ability to bind specific leukocyte subsets or to be secreted across the mucosa [2–4]. In addition to its high but transient expression in B cells during the germinal center reaction, low but biologically active amounts of AID have been detected in developing B cells, although the significance of this finding is unclear [5–7]. Besides physiologically targeting antibody genes, AID is capable of considerable collateral genomic damage. This includes mutations and DNA breaks at cancer genes, which predispose them to participate in lymphoma-associated chromosome translocations [8–11]. Hence, not surprisingly, many layers regulating AID expression and activity are in place to limit this enzyme’s potential to initiate cancer (reviewed in 11).

Beyond B cells, AID expression has been reported in some epithelial and pluripotent tissues (reviewed in 12). However, most of these observations were in tumors or *in vitro* cultured cells, leaving unresolved whether under physiologic conditions AID is expressed in non-lymphoid cells. We used genetic fate mapping (also referred to as “lineage tracing” [13]) and a transgenic reporter to explore AID expression in the mouse. We discovered that, in addition to lymphocytes, AID expression tags distinct non-lymphoid cell populations.

## Results

### Fate mapping of AID expressing cells

**Figure 1:**
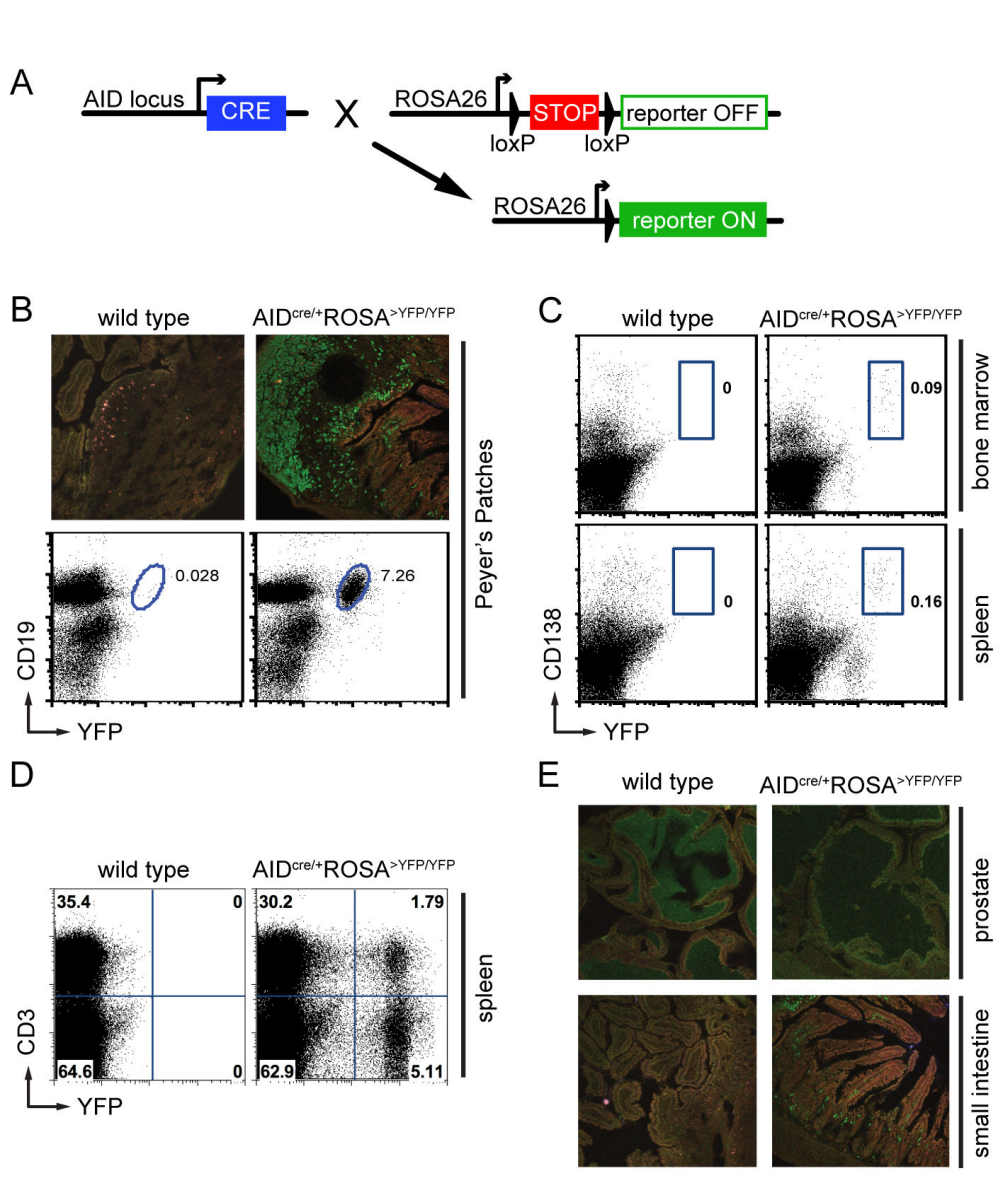
Fate mapping identifies known AID-expressing cells. **A**. Schematic representation of the genetic elements used for fate mapping. Cre-mediated excision of a loxP-flanked transcriptional stop allows for reporter expression in AID-expressing cells and their descendants. **B**. YFP expression in Peyer’s Patches by immuno-fluorescent staining (top) and flow cytometry (bottom). YFP^+^ cells are positive for the B cell marker CD19. Numbers are the percentage of cells within the shown gate. **C**. Flow cytometry identifies YFP ^+^ CD138^+^ post-germinal center plasma cells in spleen and bone marrow. **D**. YFP ^+^ CD3^+^ T cells are identified in 8 months old mice. **E**. Absence of YFP^+^ epithelial cells after immuno-staining of prostate or small intestine. The YFP^+^ cells in the small intestine are B lymphocytes and plasma cells, which are normally present in the lamina propria located beneath the mucosal epithelium.

In fate mapping, conditional and irreversible activation of a reporter allows the identification of cells that express (or have expressed) the gene of interest, as well as of their descendants [13]. To examine AID expression in the whole organism, we bred the AID^cre^ knock-in allele (in which exon 1 of the endogenous *Aicda* gene is replaced by Cre recombinase [8]) to transgenic mice conditionally expressing a reporter (Yellow Fluorescent Protein (YFP), LacZ (GNZ), or tdTomato (TOM)) driven by the ubiquitous ROSA26 promoter (ROSA^>YFP^, ROSA^>GNZ^, and ROSA^>TOM^ mice, respectively [14–16]). In double-mutant mice, Cre-mediated excision of a loxP-flanked transcriptional stop leads to permanent reporter expression, marking cells expressing (or having expressed) AID and their progeny (Figure 1A). In agreement with the known role of AID in germinal center B cells, YFP expression was readily detected in Peyer’s Patches B cells from AID ^cre/+^ ROSA^>YFP/YFP^ mice by immuno-fluorescence staining and flow cytometry (Figure 1B), as well as in post-germinal center plasma cells in the bone marrow and spleen (Figure 1C). Consistent with a recent report [17], YFP signal was also detected in a fraction of T lymphocytes of older mice (Figure 1D), but not in prostate or small intestine epithelial cells (Figure 1E). We conclude that this strategy accurately identifies known AID-expressing cell populations and their descendants.

### AID during development

**Figure 2:**
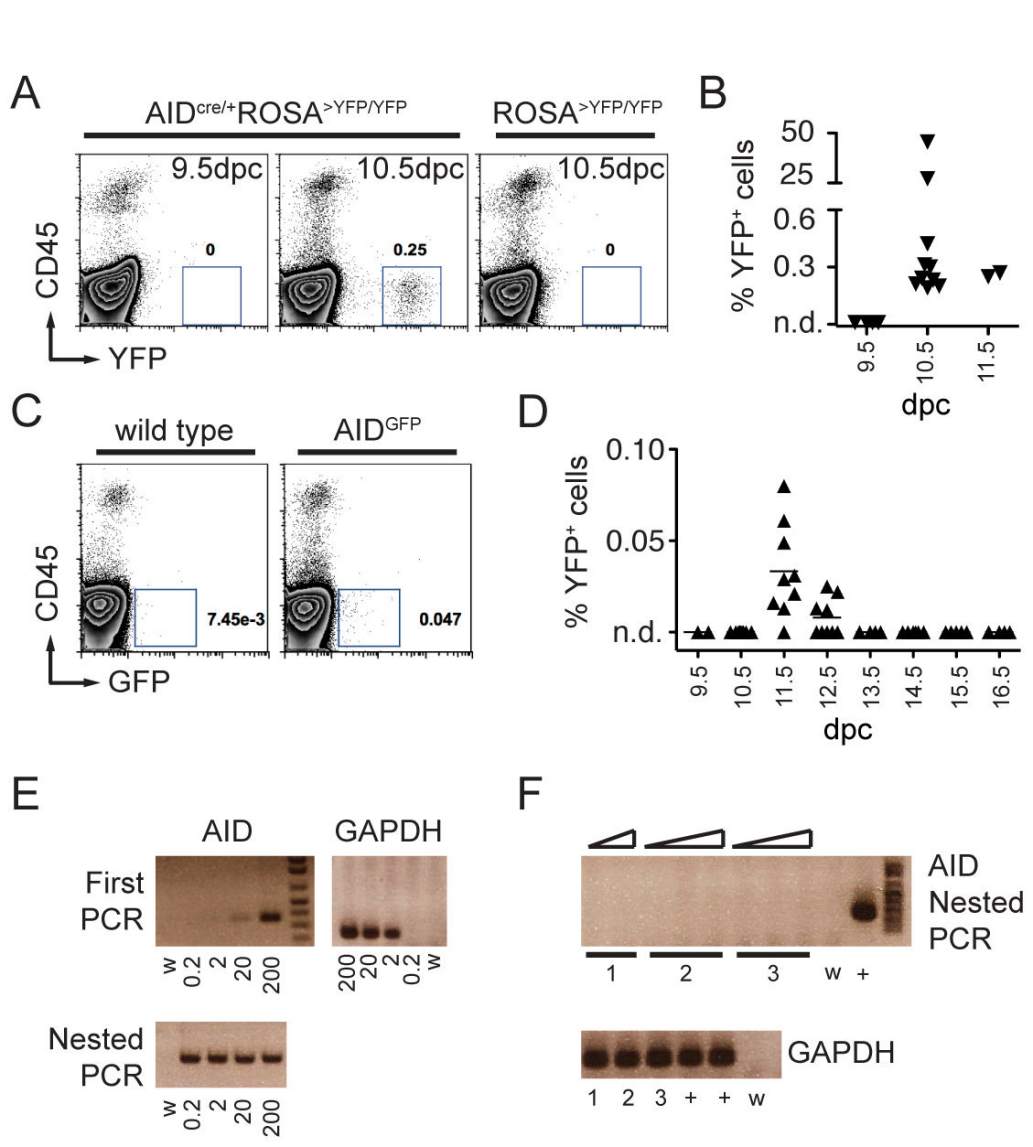
AID during development. **A**. Representative FACS plots of whole embryos revealing YFP ^+^ CD45^-^ cells starting at 10.5 dpc. Numbers are the percentage of YFP^+^ cells in the shown gate. **B**. Summary dot plot with the percentage of YFP ^+^ CD45^-^ cells in AID^cre/+^ ROSA^>YFP/YFP^ embryos at the indicated days post conception (dpc). Each triangle represents an individual embryo. n.d. is non-detectable over control. **C**. Representative FACS plots showing AID^GFP+^ cells at dpc 11.5. **D**. Summary dot plot with the percentage of GFP^+^ cells in AID^GFP^ embryos at the indicated developmental stages (days post conception). Each triangle represents an individual embryo. For dpc 9.5-12.5, cells from the whole embryo were analyzed; for dpc 13.5-16.5 analysis was limited to the upper body half. n.d. is non-detectable over wild type control. **E**. Ethidium bromide stained agarose gels with RT-PCR products obtained from sorted AID^GFP+^ germinal center B cells from Peyer’s Patches (positive control). Numbers represent cell equivalents, w is water control. GAPDH control is shown alongside. **F**. RT-PCR products from sorted AID^GFP+^ cells at 11.5 dpc from three independent experiments (lanes 1, 2, and 3). Triangles represent increasing amounts of template cDNA. Cell equivalents for the AID nested PCR were: 10, 20 (experiment 1); 5, 10, 50 (experiment 2); 1, 2, 10 (experiment 3). + is positive control and w is water. Cell equivalents for GAPDH were 10, 10, and 5 for experiments 1, 2, and 3 respectively.

To determine if AID is expressed during embryonic development, we performed timed breeding and assayed by flow cytometry single cell suspensions of whole AID ^cre/+^ ROSA^>YFP/YFP^ embryos. While no YFP^+^ cells were detected at 9.5 days post conception (dpc; n = 4), a distinct YFP^+^ population was detected in all embryos starting on dpc 10.5 (n = 10; Figure 2A and 2B). YFP^+^ cells were negative for the pan-leukocyte marker CD45 (Figure 2A). We conclude that during development AID^cre^ labels non-lymphoid cells and is expressed as early as at 10.5 dpc.

To confirm this finding, we used a different reporter system and analyzed AID^GFP^ BAC transgenic embryos, where the gene for Green Fluorescent Protein is fused in frame to exon 5 of the AID coding sequence [18]. Unlike fate mapping, the expression of GFP in this system marks only cells with ongoing AID expression [18]. Analysis of whole embryos reproducibly identified a small fraction of GFP ^+^ CD45^-^ cells, but only at 11.5 (mean = 0.03%) and 12.5 dpc (mean = 0.008%; Figure 2C and 2D). Finally, to determine if endogenous AID mRNA was produced, we sorted GFP ^+^ CD45^-^ cells from embryos at 11.5 dpc. Transcription of endogenous AID was readily detectable in control GFP^+^ sorted germinal center B cells, with a sensitivity of 20 cell equivalents (after the first PCR reaction) or 0.2 cell equivalents (upon nested PCR reaction; Figure 2E). However, we were unable to detect endogenous AID transcripts in GFP^+^ sorted cells from 11.5 dpc embryos (Figure 2F). We conclude that, if any, small amounts of AID are transiently expressed by a small fraction of non-lymphoid cells during embryonic development.

### Skin changes in AID ^cre/+^ Pten^lox/lox^ mice

**Figure 3:**
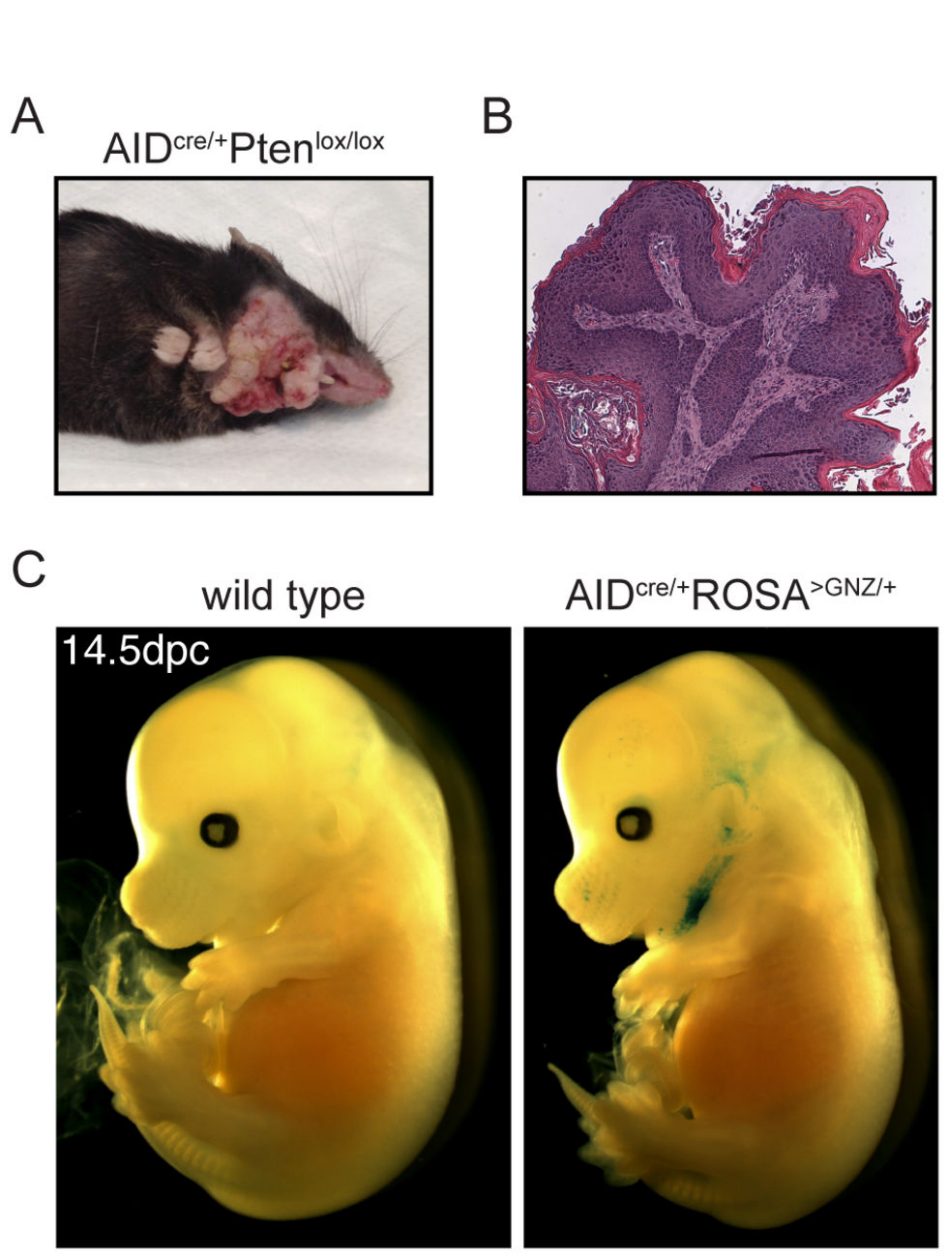
Papillomas in AID ^cre/+^ Pten^lox/lox^. Macroscopic (**A**) and histologic (**B**) appearance of papillomas in a representative 8 months old AID ^cre/+^ Pten^lox/lox^ mouse. **C**. β-galactosidase staining of whole AID ^cre/+^ ROSA^>GNZ/+^ embryos at dpc 14.5 reveals a signal (blue) in the submandibular region. Similar results were obtained at dpc 15.5 (not shown).

We reasoned that conditional deletion of a tumor suppressor gene by AID^cre^ could cause tumors originating from the AID^cre^-expressing cells, and that analysis of such tumors could in turn provide useful information on the nature of the AID expressers. We therefore crossed AID^cre/+^ to p53^lox/lox^ or Pten^lox/lox^ mice, and monitored them for tumor development. B cell lymphomas developed in 5 out of 9 AID ^cre/+^ p53^lox/lox^ mice, in agreement with the known expression of AID by B cells and the established role of the p53 tumor suppressor in lymphomagenesis (not shown and [19]). In contrast, all of ten AID ^cre/+^ Pten^lox/lox^ mice developed submandibular hair loss and skin thickening starting at 2 months of age. These changes evolved into tumors requiring euthanasia between 6 and 8 months of age (Figure 3A), and were not observed in any of the AID^cre/+^ or Pten^lox/lox^ littermate controls (n > 5; observation > 8 months). Histologically these lesions were consistent with  squamous papillomas (Figure 3B). Multifocally there were areas of follicular atypia, including hypertrophy, hyperplasia, and occasional dysplasia (not shown). Consistent with these findings, macroscopical analysis of β-galactosidase stained AID ^cre/+^ ROSA^>GNZ/+^ embryos at dpc 14.5 revealed a signal in the submandibular region (n = 12, Figure 3C). We conclude that AID^cre^-mediated deletion of a tumor suppressor gene causes B cell tumors (p53) or skin papillomas (Pten).

### AID^cre^ tagging in the central nervous system

**Figure 4:**
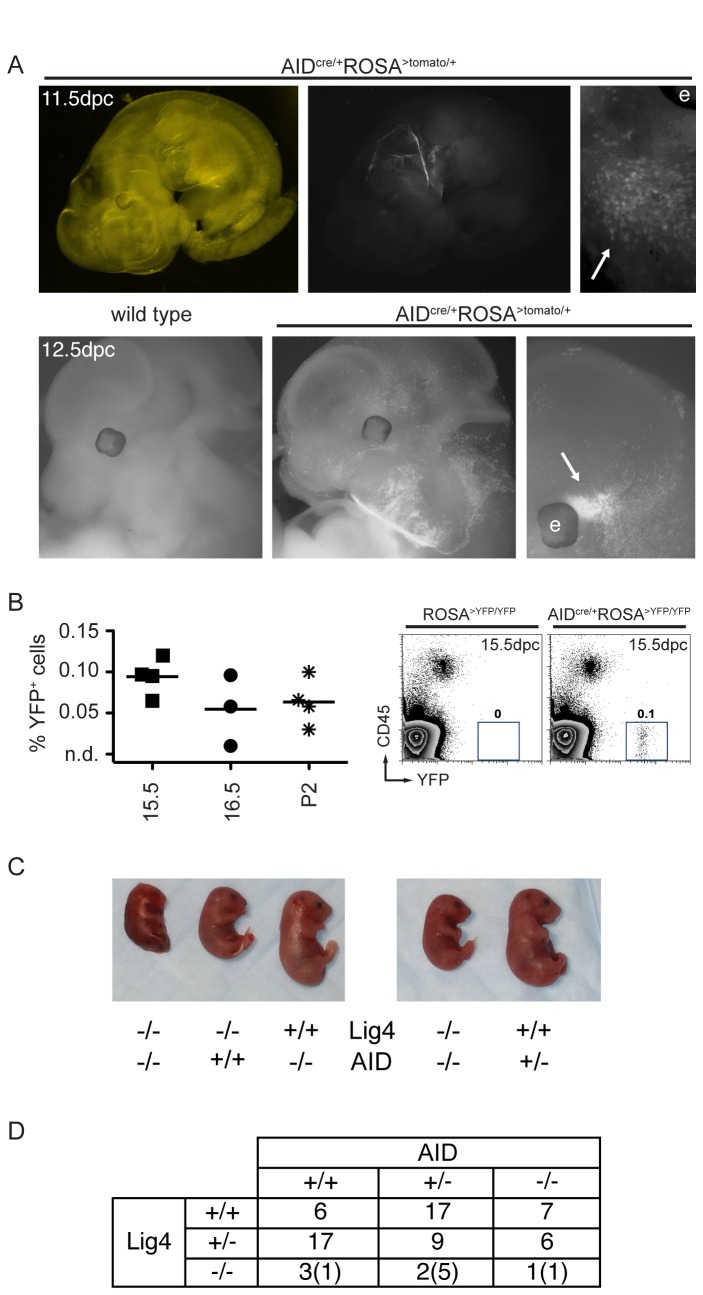
Mapping AID-tagged cells during development. **A**. Fluorescence imaging of partially cleared AID ^cre/+^ ROSA^>TOM/+^ embryos reveals a signal within the head (arrows). The letter “e” indicates the location of the eye in the higher magnification images. Top and bottom panels represent embryos at dpc 11.5 and 12.5, respectively. All images display tdTomato signal, with the exception of the top left panel, which is phase contrast of the image to its right. **B**. Left: Summary dot plot with the percentages of CD45^-^YFP^+^ cells in dissected brains from AID ^cre/+^ ROSA^>YFP/YFP^ embryos at different days post conception. P2 is postnatal day 2. Each symbol represents an independent brain. n.d. is non-detectable over control. Right: Representative FACS plots of dpc 15.5 dissected brains. Numbers are the percentage of YFP^+^ cells in the shown gate. **C**. Macroscopic appearance of Lig4^-/-^AID^-/-^ embryos (18.5 dpc) alongside controls. **D**. Table summarizing the genotypes of 75 embryos (18.5 dpc) obtained from the intercross of Lig4^+/-^AID^+/-^ parents. Eleven litters were analyzed in total. In parenthesis is the number of embryos hemorrhagic or partially resorbed at the time of analysis. DNA from 6 additional embryos was degraded and genotyping could not be performed.

To comprehensively determine the anatomical location of AID-tagged cells, we next analyzed AID ^cre/+^ ROSA^>TOM/+^ embryos at dpc 11.5 (n = 4) and 12.5 (n = 4), which had been cleared by Sca*l*e, a procedure that renders tissues optically transparent while preserving fluorescent signal [20]. Microscopic analysis confirmed tagging in the submandibular region, while no signal was detectable in the area corresponding to the genital ridges (Figure 4A and data not shown; see Discussion). In addition, we identified fluorescently labeled cells within the head (Figure 4A, arrows). To determine if tagged cells were present in brains of AID ^cre/+^ ROSA^>YFP/YFP^ embryos, we then dissected and processed this organ for flow cytometry. YFP ^+^ CD45^-^ cells were readily detected at dpc 15.5 (mean = 0.09%), 16.5 (mean = 0.05%), and postnatal day 2 (mean = 0.06% of total brain cells; Figure 4B). Hence, AID^cre^ tags non-lymphoid cells in the embryo’s brain.

AID physiologically induces DNA double-strand breaks in dividing B cells activated to undergo class switching. These breaks occur in the G1 phase of the cell cycle, and are repaired by the Non-Homologous End Joining (NHEJ) repair pathway. Accordingly, CSR in B cells is strongly impaired by the absence of the core NHEJ components Lig4 or XRCC4, leading to genomic instability and apoptosis [21]. Intriguingly, Lig4^-/-^ and XRCC4^-/-^ mice succumb to late embryonic lethality, a phenotype associated with growth delay and neuronal death starting at 10.5 dpc (see Discussion and [22–25]). Since this is the approximate timing of AID reporter expression (Figure 2), we hypothesized that AID may be responsible for the DNA damage leading to the Lig4 knockout phenotypes. In order to test this possibility, we generated Lig4^+/-^AID^+/-^ mice, intercrossed them to each other, and analyzed the progeny at 18.5 dpc. As previously reported, Lig4^-/-^ embryos were less frequent and smaller, but this was independent of their AID status (Figure 4C and 4D). We conclude that AID deficiency does not rescue the growth delay or embryonic lethality of Lig4 knockout mice.

**Figure 5:**
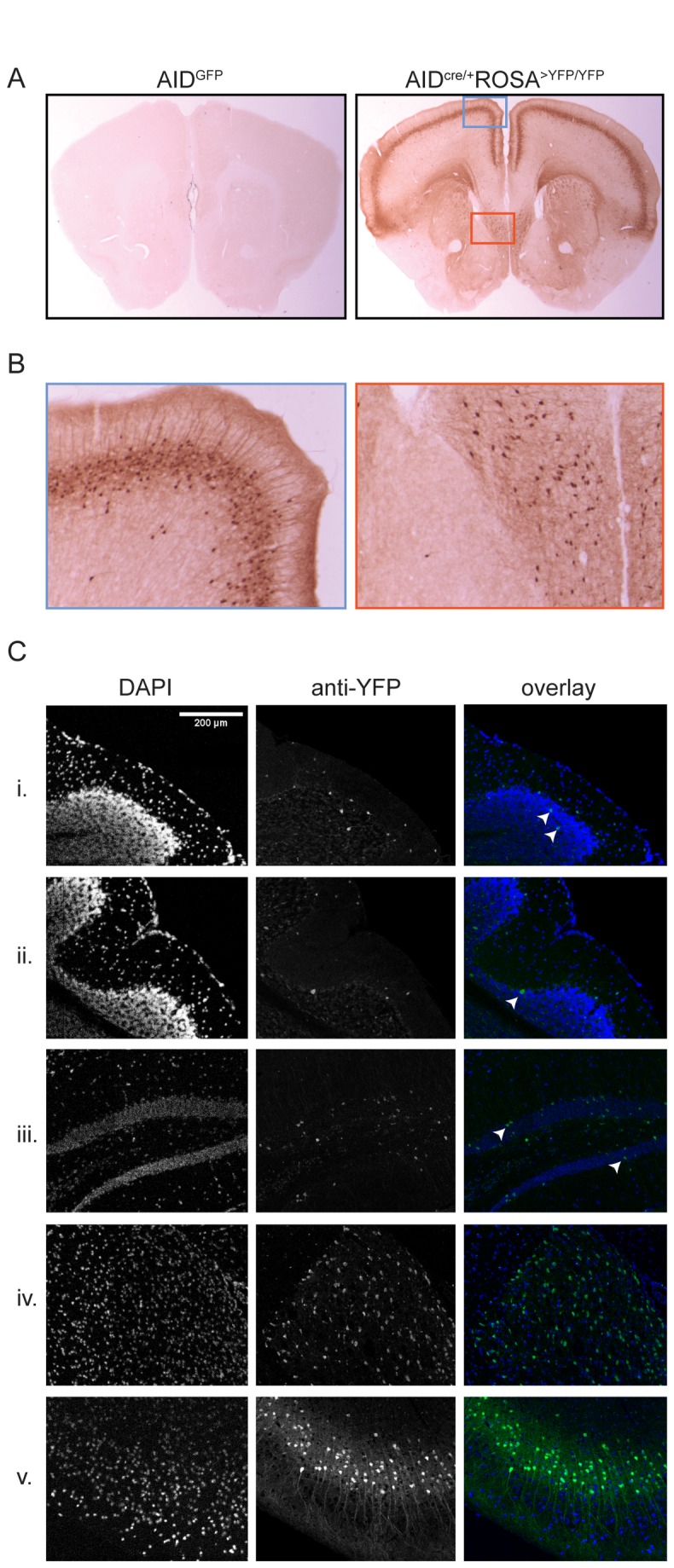
AID in the adult mouse brain. **A**. Immuno-histochemical detection of GFP/YFP in the brains of 8 weeks old mice. Shown is coronal section 25 (out of 71, starting from rostral). **B**. Higher magnification of the boxed areas in panel A demonstrating expression in pyramidal layer IV (left) and septal area (right). **C**. Immuno-fluorescence images of sagittal sections of AID ^cre/+^ ROSA^>YFP/YFP^ brains showing reporter staining in scattered granule (i) and Purkinje (ii) cells in cerebellum, and granule cells in the dentate gyrus (iii). More uniform staining is observed in the antero-ventral nucleus of thalamus (iv) and cortex layer IV (v).

Finally, to determine the nature and localization of AID^cre^-tagged cells in the brain we immuno-stained coronal sections of adult mouse brains. While GFP signal was undetectable in AID^GFP^ brains, a distinct pattern was detected in AID ^cre/+^ ROSA^>YFP/YFP^ (Figure 5A). In cortex, YFP^+^ labeling was specific for pyramidal cells in layer IV. The lateral septal area and the anterior thalamus were also enriched for YFP^+^ cells (Figure 5B and not shown). We observed mosaic expression in the rest of the brain, with a small fraction of positive cells in most of the other anatomical areas. For example, in the cerebellum, where cell identity can be recognized from anatomical position, few stained cells were observed within granule, Purkinje and molecular layers (Figure 5C). We conclude that, strikingly, AID^cre^ labels pyramidal layer IV neurons, as well as subsets of other cell types in the brain.

## Discussion

AID is responsible for targeted DNA mutations (SHM) and deletional recombinations (CSR) at the immunoglobulin heavy chain gene of mature B lymphocytes [2–4]. Besides being transiently expressed at high levels in B cells during the germinal center reaction, low levels of AID have been reported in developing B cells and in a fraction of T cells [5–7,17]. Whether other cell types express AID under physiologic conditions is less clear. Using fate mapping, a sensitive technique based on genetic labeling, we identified AID in a small fraction of non-lymphoid embryonic cells. This finding was supported by a second, independent genetic reporter, which tracks cells with ongoing AID expression. However, we were unable to confirm expression of endogenous AID mRNA by sensitive RT-PCR methods, suggesting that, if at all, AID is expressed only transiently and at very low levels in embryonic cells. Of note, AID transcripts in lymphocytes have a half-life of only one hour [26]. Given that cytidine deamination by AID is not a very efficient reaction [27,28], this raises the possibility that such low levels may be insufficient for significant biological activity.

Mutations at p53 and Pten are common in cancer. p53 mutation is frequent in human lymphoid neoplasia, and in mouse models p53 deficiency synergizes with AID in causing B cell lymphoma [9,29]. Accordingly, B cell tumors were predicted in AID ^cre/+^ P53^lox/lox^ mice. In contrast, the development of skin changes and papillomas in AID ^cre/+^ Pten^lox/lox^ mice was unexpected. While the role of Pten in papillomatogenesis is known from Cowden’s disease patients and mouse models [30,31], the focal nature of the AID^cre^-induced changes was a surprise, and revealed a very specific pattern of expression confined to the submandibular skin. This finding was further confirmed by β-galactosidase staining of embryos. Hence, lineage-specific deletion of tumor suppressors is a useful genetic approach for revealing tissues expressing a gene of interest.

Intriguing similarities exist between the nervous and immune system [32]. One commonality is the critical role of intact NHEJ in both neurons and lymphocytes. In the nervous system mutation or deletion of the NHEJ core components Lig4 or XRCC4 causes microcephaly, neuronal death during development, and promotes medulloblastomas with recurrent chromosome alterations [33–36]. In the immune system the same deficiency is responsible for immune defects due to impaired VDJ and CSR in B cells, genomic instability, and lymphoma with translocations [21,23,24,37]. In B cells undergoing CSR, AID initiates programmed DNA double-strand breaks in the G1 phase of the cell cycle, which require NHEJ for repair. The source of DNA damage in developing post-mitotic neurons that requires NHEJ is less clear [36]. Since AID^cre^-tags neurons, and tagging coincides with the developmental stage when neuronal death is observed in NHEJ-deficient embryos, we tested genetically the possibility that AID may be the source of DNA damage, which is associated with growth retardation and embryonic lethality. Lig4/AID double-mutant embryos were however indistinguishable from control, leaving the source of damage undetermined.

AID has been proposed to play a role in the process of active DNA cytosine demethylation (reviewed in 38). During mammalian development, AID may regulate methylation in pluripotent tissues (embryonic stem cells, primordial germ cells, and oocytes [39]), while in *Danio rerio* AID demethylates artificial DNA fragments introduced in one-cell embryos [40]. Interestingly, in mouse primordial germ cells, the extensive epigenetic reprogramming resulting in global demethylation takes place at day 11.5 of development [41], which coincides with the timing of AID detection using our reporter systems (Figure 2). However, no reporter signal was detected in the genital ridges of cleared embryos at dpc 11.5, consistent with the previously reported absence of AID transcripts in this tissue [42]. Moreover, crossing AID ^cre/+^ ROSA^>YFP/YFP^ to ROSA^>YFP/YFP^ mice results in only a fraction of YFP^+^ embryonic cells, rather than 100% positive cells as would be expected if AID were expressed in the germline. Hence, while our data do not speak to the issue of demethylation, they do not support expression of AID in the mouse germline.

In the central nervous system, AID-tracing labels distinct cell types in cortex and thalamus as well as sporadic cells throughout the brain. This suggests that AID expression is regulated by developmental cues in some cell types, and stochastic in others. The sporadic AID-tagging we observed in cerebellum is reminiscent of the pattern of retrotransposon reporters in mouse brain [43]. Considering that in B cells AID can be induced by retroviral infection [5], it is tempting to speculate that in neuronal cells it may provide a protective mechanism against the potentially deleterious action of retrotransposition, which takes place in neuronal stem cells. The functional significance of the uniform expression in cortex layer IV is unclear. Pyramidal layer IV cells extend the apical dendrite into layers II/III and have been implicated in signal transduction both between layers and cortical columns [44].

In summary, we present evidence for AID expression in non-lymphoid cells during mouse development. The biological function of AID in these tissues remains to be determined.

## Materials and Methods

Mice. AID^cre^ (Aicda^tm1(cre)Mnz^ [8]), AID^GFP^ [18], ROSA^>YFP^ (B6.129X1-Gt(ROSA) 26Sor^tm1(EYFP)Cos^/J [14]), ROSA^>GNZ^ (B6; 129-Gt(ROSA) 26Sor^tm1Joe^/J [15]), ROSA^>TOM^ (B6; 129S6-Gt(ROSA) 26Sor^tm14(CAG-tdTomato)Hze^/J [16]), Pten^lox/lox^ (B6.129S4-Pten^tm1Hwu^/J [45]), p53^lox/lox^ (B6.129P2-*Trp53* ^*tm1Brn*^/J[46];, AID^-/-^ [47], and Lig4^-/-^ (B6; 129S6-Lig4^tm1Fwa^/Kvm [22]) mice were all previously described. Experiments were in accordance with protocols approved by the Rockefeller University Institutional Animal Care and Use Committee (#09013 and #10027).

### Immuno-staining of adult mouse tissues

Tissues were fixed in paraformaldehyde for one hour and 30% sucrose for overnight prior to embedding in OCT medium. Small intestine and prostate were stained with Alexa-488 conjugated anti-GFP/YFP antibodies (Invitrogen, clone A21311) as previously described in detail [48]. Brain sections were stained similarly as described above for the immuno-fluorescence and by Neuroscience Associates for the immuno-histochemistry (Knoxville, TN).

### Flow cytometry and sorting

Single cell suspensions of lymphoid tissues were stained with labeled antibodies to CD3e (eBioscience, clone eBio500A2), CD19 (eBioscience, MB19-1), and CD138 (BD Pharmingen, 281-2). Whole mouse embryos or dissected brains were processed into single cells using the trypsin-based Neural tissue dissociation kit T (Miltenyi Biotec). Staining was with fluorophore-conjugated antibodies against CD45 (BD Pharmingen, 104). Samples were acquired on a FACSCalibur (BD) and analyzed with Flowjo (Treestar). AID^GFP+^ cells were sorted directly into TRIzol LS on a FACSAria (BD).

### RT-PCR

RNA was extracted from TRIzol LS, precipitated in the presence of glycogen, and reverse transcribed using SuperscriptIII according to the manufacturers’ protocols. Crossintronic primers specific for endogenous AID mRNA and amplification conditions were as follows. First PCR reaction: 5-CCCGGCACGTGGCTGAGTTTC-3 and 5-ATCACGTGTGACATTCCAGGAG-3 (56C annealing temperature, 1 minute extension time, 35 cycles). One µl from the first PCR reaction was used as template for the nest: 5-CTGAGATGGAACCCTAACCTCAGCC-3 and 5-AAGTCATCGACTTCGTACAAG-3 (54C, 1 minute, 35 cycles). GAPDH was amplified with 5-TGAAGCAGGCATCTGAGG-3 and 5-CGAAGGTGGAAGAGTGGGAG-3 (55C, 1 minute, 35 cycles).

### Embryo fixation, LacZ staining, and clearance

Embryos were washed twice with PBS and fixed with 4% PFA for 2.25 hours at 4°C. For LacZ staining, after two washes with washing buffer (PBS, 2mM MgCl_2_, 0.01% deoxycholic acid, 0.02% Igepal CA-630) for 45min each, embryos were incubated with staining solution (washing buffer supplemented with 1mg/ml X-gal, potassium ferricyanide 5mM, potassium ferrocyanide 5mM) at 4°C in the dark for 5.5 hours. The reaction was stopped by replacing the staining solution with washing buffer. For fluorescent imaging of tdTomato, embryos were washed with PBS, fixed with 4% PFA for 1 hour, then washed again in PBS for 1h. The clearance was performed with the Sca*l*e A2 reagent [20]. Embryos were kept in Sca*l*e A2 at 4°C and the solution was replenished after its color changed.
